# Hemorrhagic septicemia in the United States: molecular characterization of isolates and comparison to a global collection

**DOI:** 10.1177/10406387251342528

**Published:** 2025-05-24

**Authors:** Kelli J. Maddock, Brianna L. S. Stenger, Heidi L. Pecoraro, Jill C. Roberts, John Dustin Loy, Brett T. Webb

**Affiliations:** Veterinary Diagnostic Laboratory, North Dakota State University, Fargo, ND, USA; College of Public Health, University of South Florida, Tampa, FL, USA; Veterinary Diagnostic Laboratory, North Dakota State University, Fargo, ND, USA; Veterinary Diagnostic Laboratory, North Dakota State University, Fargo, ND, USA; College of Public Health, University of South Florida, Tampa, FL, USA; Nebraska Veterinary Diagnostic Center, University of Nebraska– Lincoln, Lincoln, NE, USA; Veterinary Diagnostic Laboratory, North Dakota State University, Fargo, ND, USA

**Keywords:** hemorrhagic pasteurellosis, hemorrhagic septicemia, *Pasteurella multocida*

## Abstract

Hemorrhagic septicemia–causing strains of *Pasteurella multocida* are endemic in Asia and Africa, but naturally occurring hemorrhagic septicemia has not been described in livestock in the United States since 1993. There are 5 capsular types of *P. multocida*: A, B, D, E, and F. Two capsular types (B, E) cause hemorrhagic septicemia, whereas capsular types A and, to a lesser extent, D are associated with enzootic bovine pneumonia. Here we describe 2 naturally occurring cases of hemorrhagic septicemia caused by *P. multocida* capsular type B:3,4 in the United States, including molecular characterization of these strains, with a comparison to available reference strains and publicly available genomes of *P. multocida* capsular type B. Genomic analyses demonstrated that our case strains are similar to a strain isolated from New Jersey cattle in 1968 and to contemporaneous strains from New Zealand and Canada. These strains are different from those circulating globally, as demonstrated by the need to assign new sequence types for our isolates. Hemorrhagic septicemia appears to be re-emerging globally in countries that have not seen outbreaks in decades and may be poised for re-emergence in the United States given the identification of these novel strains.

Hemorrhagic septicemia (HS) is an endemic disease of bovids in Asian and African countries, but is considered a foreign animal disease that is reportable to the USDA in the United States.^42^ HS causes rapid and significant death loss if left untreated in affected herds.^15^ Grossly, cattle typically have edematous neck and brisket regions and pericarditis or polyserositis.^15,44^

*Pasteurella multocida*, the causative agent of HS, is a facultatively anaerobic gram-negative coccobacillus in the *Pasteurellaceae* family. There are 5 capsular types: A, B, D, E, and F.^8,9,30^ Each capsular type is generally associated with a particular host animal species. Capsular types A, D, and F can cause disease in a number of animal species and humans.^6,43^ Bovine enzootic pneumonia is most commonly associated with *P. multocida* capsular type A and, to a lesser extent, capsular type D.^43^ Capsular types B and E cause HS in domestic cattle (*Bos taurus*), bison (*Bison bison*), and buffalo (*Bubalus bubalis*), and may have wildlife reservoirs.^7,15,24^

*P. multocida* strains are further characterized by their lipopolysaccharide (LPS) serovar, classically identified as Heddleston serovars 1–16^5,19^ or genomically by loci L1–L9.^18,36^ Strains are designated capsular:serovar (e.g., A:3 or B:2,5). HS-causing strains are traditionally B:2,5 or E:2,5 (genotype L2). Cases of HS caused by B:3,4 (genotype L3) have been described in cattle (New Jersey)^23,31^ and bison (Montana)^31^ in the United States, beef calves in Canada^16^ and New Zealand,^27^ and fallow deer (*Dama dama*) in Denmark^1^ and the United Kingdom.^29^ In North America, cases of B:2,5 HS were confirmed in Wyoming bison in 1922^31^ and in beef calves in California in 1993^4,31^ and Canada in 2020.^16^ Cases of B:1 HS in cervids have been reported in North Carolina, Montana, and Wyoming.^29^

Historically, strain typing of *P. multocida* has required capsular and LPS typing sera, which are cumbersome to produce and maintain for the 5 capsular and 16 somatic serovars. As of 2001, only 2 laboratories were known to maintain the reagents for capsular serotyping.^40^ The World Organisation for Animal Health (WOAH, OIE) Terrestrial Manual lists serologic and PCR assays as suitable methods for capsular and LPS typing of *P. multocida*, as well as other HS strain–specific PCR assays.^44^ The capsular and LPS PCR methods are reliable alternatives to conventional serologic methods; however, 6 capsular primer sets^40^ and 8 LPS primer sets^18^ are required for a complete typing panel, making the process impractical for routine diagnostic use. L9 was only described in 2024, so PCR primer sets are not available.^36^ In the age of genomic testing, sequence-based capsular and LPS typing algorithms have been introduced and appear to be reliable tools for typing.^11^ A matrix-assisted laser desorption/ionization–time-of-flight mass spectrometry (MALDI-TOF MS) biomarker–based model was developed as a potential tool for identifying HS-causing strains in any laboratory possessing the technology.^25^ We used some of the strains analyzed in our study to develop the MALDI-TOF MS biomarker–based model for differentiating HS-causing strains (capsular types B and E) from non-HS strains (capsular types A, D, and F) of *P. multocida*.^25^ The method can be utilized to rapidly screen strains prior to performing more costly and time-consuming molecular workups.

Historically, HS may have been confused with other diseases and was possibly an overused term.^2,10^ Confirmed outbreaks of HS occurred in the United States as early as 1922.^17,31^ HS was last described in the United States in beef calves in California in 1993^4,31^; however, the last outbreak noted on the 2018 WOAH (OIE) Reportable Disease report^42^ indicates that HS last occurred in domestic cattle in a New Jersey dairy herd in 1968.^23^ HS is classically associated with bovids, but there are also reports of HS in wild and domestic pigs.^41^ Wolves (*Canis lupis*) were suggested as possible vectors of capsular type B HS in Germany,^24^ and a 2021 study reported capsular type B *P. multocida* isolated from chicken droppings.^33^

Here we describe 2 cases of *P. multocida*–associated HS in bovids from the United States. Because HS has not been reported in the United States for decades, we feel it is important to describe diagnostic cases of HS for new generations of veterinarians and microbiologists and to remind diagnosticians of this potentially re-emerging disease. In addition, we performed genomic analyses on whole-genome sequences of strains to determine the relatedness of the case strains to historical isolates as well as those circulating globally. These sequences could also provide additional epidemiologic or host-based information on the origin and reservoirs of these strains, given a paucity of identification of such *P. multocida* strains over many decades.

## Materials and methods

### Cases

Case 1 was a 5-mo-old Black Angus heifer calf from a herd of 150 commercial cattle. This animal was 1 of 2 calves that had been found dead following a 24–48-h period of unilateral hindlimb lameness. Case 2 was a 2.5-mo-old bison bull calf that was found dead without any clinical signs being noticed. This animal was one of 6 calves that were recently found dead over the course of a week from a herd of unknown size.

Both animals were submitted to the North Dakota State University–Veterinary Diagnostic Laboratory (Fargo, ND, USA) for routine autopsy in 2020 (case 1) and 2022 (case 2). The cases originated in the upper Midwest. Fresh tissues were submitted for routine bacterial culture as described below. Formalin-fixed paraffin-embedded tissues were routinely processed, sectioned, and H&E stained for review by 2 veterinary anatomic pathologists.

### Bacterial culture

Pericardium and lung from case 1 and lung from case 2 were submitted for routine bacterial culture. Each tissue was inoculated onto trypticase soy agar with 5% sheep blood (SBA) (Remel), chocolate agar (Remel), MacConkey agar (Remel), and *Mycoplasma* agar (University of California–Davis Biological Media Services) and incubated at 37°C for 24 h in 5% CO_2_. We used 4 lyophilized reference strains of *P. multocida*, obtained from the National Veterinary Services Laboratory (Ames, IA, USA). The conventional capsular and LPS serovars, along with animal species, year of isolation, and country of origin of these reference strains, were also provided. Lyophilized strains were reconstituted with 1 mL of brain-heart infusion broth (Hardy) and inoculated onto SBA and incubated at 37°C for 24 h in 5% CO_2_. Pure culture strains were identified to the species level using the manufacturer-recommended direct spot method with α-cyano-4-hydroxycinnamic acid matrix (Bruker) overlay and a standard MALDI-TOF MS database (Bruker). Antimicrobial susceptibility testing was performed using commercial BOPO7F plates (Sensititre), per the manufacturer’s recommendations, and incubated for 18–24 h at 35°C in ambient conditions. Antimicrobial susceptibility test results were automatically read (BIOMIC V3; Giles) using interpretations from the Clinical and Laboratory Standards Institute standards contemporaneous to the time of testing, VET08 4th and VET01S 5th editions, respectively.^12,13^

Pure culture case and reference strains were subcultured onto SBA (Remel) and incubated at 37°C for 24 h in 5% CO_2_. The strains were then passed to fresh SBA plates 2 more times under the same conditions. A heavy suspension of each bacterium was suspended in nuclease-free water for downstream sequencing applications.

### Molecular methods

Nucleic acids were extracted (KingFisher Flex, ThermoFisher; standard MagMAX CORE program and extraction kit, Applied Biosystems) according to the manufacturers’ instructions for 200-μL sample input volumes. Capsular typing PCR was performed as a screening tool prior to sequencing using primer sets described previously.^40^ In short, each 25-µL reaction consisted of 10 µL of PerfeCta SYBRGreen FastMix, Low ROX (Quantabio), 1 µL of each primer (10 µM), and 5 µL of template DNA. PCR was performed (Mini Amp Plus; ThermoFisher). Cycling conditions consisted of an initial denature step of 95°C for 5 min and 30 cycles of 95°C for 30 s, 55°C for 30 s, and 72°C for 30 s. Amplified products were visualized on 3% agarose gel (EZ-Vision Three; VWR).

Sample extracts were subjected to library preparation (DNA Prep kit; Illumina) and half-reagent volumes as described previously.^3^ Samples were barcoded to multiplex. Whole-genome sequencing was then performed (iSeq 100 or MiSeq; Illumina) using standard cartridges to generate 150-bp paired reads. Reads were trimmed at both ends (FASTQ Toolkit v.2.2.6; BaseSpace). Reads from each library were assembled via de novo assembly (SPAdes v.3.9.0; BaseSpace). The assembled genomes were quality control checked (DRAGEN FastQC and MultiQC v.3.9.5; BaseSpace) to ensure >30× coverage. Genomes were deposited in the National Center for Biotechnology Information (NCBI) database^34^ under BioProject PRJNA1124145.

### GenBank accessions

Additional reference genomes were included in the sequence analysis to add diversity to the sequence analysis set. On 2024 March 2, the BLAST database (https://blast.ncbi.nlm.nih.gov/Blast.cgi) was queried using *bcbD* primers specific to capsular type B *P. multocida*^40^ with “Standard databases”, organism set to “*Pasteurella multocida* (taxid:747)”, and “Highly similar sequences (megablast)” settings selected. For an accession to be included in the analysis, it must: 1) have an E value of <1; 2) have a 100% query cover and percent identity to the forward and reverse capsular type B *bcbD* primers; and 3) be a complete genome of *P. multocida*. A total of 11 complete genomes of *P. multocida* matching the search criteria were identified in the NCBI database: CP017961, CP052764, CP052765, CP066223, CP072655, CP092967, CP133836, LR134532, CP048402, CP133809, and CP133810.

### Sequence analysis

The assembled FASTA files for each case, reference, and NCBI accession (*n* = 17) were subjected to capsular and LPS serovar prediction via the “*P. multocida* sequence database for prediction of capsular type and LPS group” database hosted by the Bacterial Isolate Genome Sequence Database (BIGSdb).^11,22^ Multi-locus sequence typing (MLST) was performed using the PubMLST database hosted by BIGSdb^22^ to query each strain against both the Multi-host and Rural Industries Research and Development Corporation (RIRDC) MLST schema for *P. multocida*.^14,39^

Reference, field, and NCBI accession genomes were further assessed for sequence similarity. Genomes were annotated (Prokka Genome Annotation, Galaxy v.1.14.6+galaxy0) with standard settings, with the exception of the following fields: “Genus name” set to *Pasteurella*, “Species name” set to *multocida*, “Kingdom” set to bacteria, and “Use genus-specific BLAST database” set to yes.^35^ Then, Prokka-generated .gff files were subjected to the Roary Pangenome Pipeline (Galaxy v.3.13.0+galaxy2) using standard settings and all file outputs selected.^28^ Finally, Roary newick files were used to generate a pangenome presence/absence matrix tree in Roary Plots (Galaxy v.1.0).

## Results

### Pathology

#### Case 1

**Figure 1.**
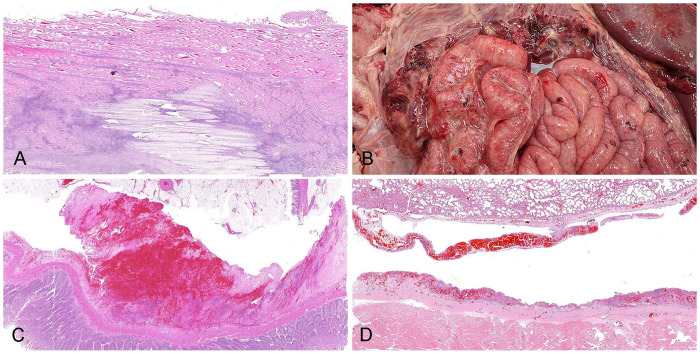
Hemorrhagic septicemia in (A) a 5-mo-old Black Angus heifer calf and (B–D) a 2.5-mo-old bison bull calf. **A.** Hemorrhagic myositis. **B.** Gross and **C**) microscopic images of hemorrhagic and fibrinous peritonitis. **D.** Hemorrhagic pleuritis (top tissue) and peritonitis (bottom tissue).

On gross examination, the 5-mo-old Black Angus heifer calf was in good body condition with mild postmortem autolysis. There was diffuse pulmonary edema and congestion, along with fibrinous effusion within the pericardial sac and fibrinous pericarditis. Scattered petechiae were present in the subcutis and on serosal surfaces of thoracic organs. In addition, multiple muscle groups of the upper left hindlimb were pale and dry and contained areas of hemorrhage. Microscopically, fibrin and suppurative inflammation, admixed with bacterial colonies, expanded the pericardium and pericardial surface. Within the left hindlimb skeletal muscle was extensive myocyte necrosis, along with numerous neutrophils and macrophages, and bacterial colonies were present in the perimysium surrounding muscle fibers (Fig. 1A). Lung culture yielded heavy growth of *P. multocida*. Lung culture for *Mycoplasma* species and PCR for bovine herpesvirus 1, bovine viral diarrhea virus, bovine respiratory syncytial virus, and bovine coronavirus were negative.

#### Case 2

On gross examination, the 2.5-mo-old bison bull calf was in good body condition with mild postmortem autolysis. Grossly, edema and hemorrhage expanded the omentum and mesentery, and fibrin strands covered the gastrointestinal tract and liver (Fig. 1B). Hemorrhages were noted within the lung lobes and along the abdominal body wall. The left stifle contained thick, fibrinous fluid. Histology confirmed fibrin and hemorrhage on the surfaces of the lung and diaphragm (Fig. 1C) and within the left stifle joint. Hemorrhage was present within the serosa and tunica muscularis of the intestines (Fig. 1D). In addition, there was mild enterocolitis with increased numbers of mixed inflammatory cells and rare crypt dilation and necrosis. Lung and intestinal cultures yielded heavy growth of *P. multocida* and mixed bacteria. Additional lung culture for *Mycoplasma* species was negative. *Clostridium perfringens* genotype A was also cultured from the intestine. Fecal flotation for parasites and PCR for bovine coronavirus, rotavirus, and *Cryptosporidium parvum* were negative.

### Bacterial strain characterization

Heavy growth of small tan-to-gray colonies predominated on blood and chocolate agar; no growth was observed on MacConkey agar. All case and reference strains were identified as *P. multocida* with scores of >2.0 by MALDI-TOF MS. The case strains were susceptible to all antimicrobials with bovine interpretations contemporaneous to the time of testing for *P. multocida* (ceftiofur, danofloxacin, enrofloxacin, florfenicol, gamithromycin, spectinomycin, tetracycline, tildipirosin, tilmicosin, and tulathromycin).

Due to the constellation of lesions in these cases and lack of typical bronchopneumonia, the strains were further evaluated. The strain from case 1 was confirmed as capsular type B at the USDA via PCR using the Townsend capsular PCR described previously.^40^ The strain from case 2 was confirmed internally as capsular type B using the same method.

### Molecular analysis

Each case and reference strain were positive for capsular type B by the *bcbD* PCR assay. Predicted capsular type based on genomic results was consistent with PCR findings; all capsular types were predicted to be type B. Cases 1 and 2, reference strain P1669, and GenBank accessions CP133809 and CP133810 were LPS genotype L3, consistent with Heddleston serovars 3,4. In contrast, reference strains P932, P4257, P1487, and the remaining GenBank accessions were LPS genotype L2, consistent with Heddleston serovars 2,5. New MLST sequences types were assigned in the PubMLST database for cases 1 and 2, and P1669. Most other strains were ST122 within the ST122 clonal complex (11 of 17) in the RIRDC scheme and ST44 (8 of 17) by the multi-host MLST scheme (Table 1).

**Table 1.** Metadata for case, reference, and GenBank strains of *Pasteurella multocida* used in our study.

Strain	Host	Country	Year	RIRDC MLST	Mh MLST	WGS cap	WGS LPS	Conventional serovar
Case								
1	Cattle	USA	2020	ST453	ST212	B	L3	NP
2	Bison	USA	2022	ST538	ST347	B	L3	NP
Reference								
Ref-P932	Bison	USA	1956	ST122 CC ST122	ST64	B	L2	2,5
Ref-P4257	Cattle	CN	1980	ST122 CC ST122	ST73	B	L2	2,5
Ref-P1487	Cattle	IN	1966	ST122 CC ST122	ST44	B	L2	2,5
Ref-P1669	Cattle	US	1968	ST543	ST348	B	L3	3,4
NCBI accession								
CP017961	Cattle	IR	1936	ST122 CC ST122	ST44	B	L2	NA
CP052764	Chicken	BD	2017	ST122 CC ST122	ST44	B	L2	NA
CP052765	Chicken	BD	2017	ST122 CC ST122	ST44	B	L2	NA
CP066223	Yak	TB	2020	ST122 CC ST122	ST44	B	L2	NA
CP072655	Yak	TB	2018	ST122 CC ST122	ST44	B	L2	NA
CP092967	Buffalo	IN	2020	ST122 CC ST122	ST44	B	L2	NA
CP133836	Cattle	CN	2022	ST122 CC ST122	ST44	B	L2	NA
LR134532	Bovine	MM	Prior to 1960	ST122 CC ST122	ST44	B	L2	NA
CP048402	Cattle	CA	2020	ST459	ST233	B	L2	NA
CP133809	Cervine	NZ	2022	ST64	ST342	B	L3	NA
CP133810	Bovine	NZ	2022	ST62	ST343	B	L3	NA

BD = Bangladesh; CA = Canada; cap = capsular type; CC = clonal complex; CN = China; GB = United Kingdom; IN = India; IR = Iran; L = Locus; LPS = lipopolysaccharide; Mh = multi-host; MLST = multi-locus sequence type; MM = Myanmar; NA = not available; NP = not performed; NZ = New Zealand; Ref = reference strains; RIRDC = Rural Industries Research and Development Corporation; ST = sequence type; TB = Tibet; USA = United States; WGS = whole-genome sequence.

**Figure 2.**
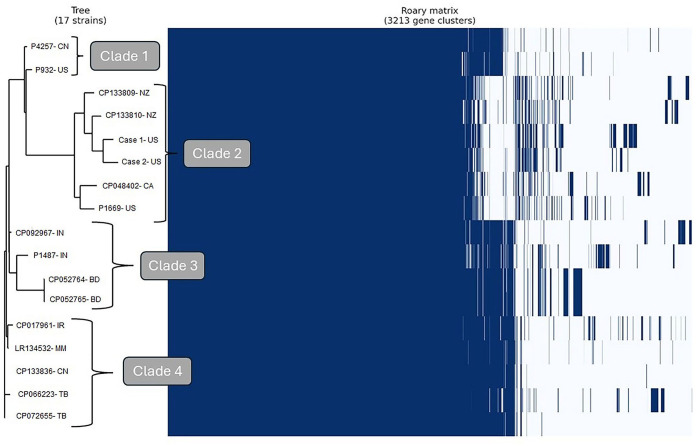
Roary pangenome genome alignment, phylogenetic tree, and clade assignments of *Pasteurella multocida* capsular type B strains analyzed in our study. BD = Bangladesh; CA = Canada; CN = China; IN = India; IR = Iran; MM = Myanmar; NZ = New Zealand; TB = Tibet; US = United States.

A core genome alignment was performed via the Roary pangenome pipeline (Fig. 2). The 17 strains included in our study shared 1,795 core genes out of 3,213 gene clusters identified. Four clades were identified in our analysis. Clade 1 was comprised of reference strains P4257 (China) and P932 (United States). Clade 2 was comprised of the strains from the United States and Canada (cases 1 and 2, P1669, CP048402) and those from New Zealand (CP133809, CP133810). Strains from India (CP092967, P1487) and Bangladesh (CP052764, CP052765) formed clade 3. Clade 4 included strains from Iran, Myanmar, China, and Tibet (CP017961, LR134532, CP133836, CP066223, CP072655).

## Discussion

The gross and histologic changes in our cases were similar to those observed in previous reports of HS in North America^16,23^ and New Zealand.^27^ Both cases had fibrinosuppurative serositis, isolated to the pericardium in case 1 but more widespread in case 2, in which the thoracic and peritoneal cavities were additionally affected. The locally extensive necrosuppurative myositis observed in case 1 mirrors the description of lesions from more recent North American cases.^16,23^ The initial presentation of lameness that was observed in case 1 has been described in both experimental infections^20^ and natural infections.^27^ The changes present in the intestine of case 2 were quite mild, and their relationship to the primary process is uncertain. Given the absence of reports of enteritis from cases of HS, it seems likely that they were preexisting undifferentiated enteritis rather than being referable to HS. Presentation of these cases within herds seemed to differ somewhat from descriptions of outbreaks in other countries due to the low number of animals affected.^15^ Further evaluation of potential differences was not possible due to the low numbers of animals received from affected herds and limited additional history. Overall, the similarity of these cases to those from the United States,^23^ Canada,^16^ and New Zealand,^27^ and resulting strain similarity, suggests that these strains could disproportionately affect younger animals.

All 17 strains included in our study shared 1,795 core genes. Clade 2, which contained our case strains, was markedly different from the other 3 clades in that strains from this clade were missing accessory genes that strains in the other clades possessed. Our case strains were characterized as B:3,4, and they clustered with reference strain P1669, which was isolated from cattle in New Jersey in 1968.^23,31^ The outlier in clade 2 is CP048402, a strain from a beef heifer calf in Alberta, Canada.^16^ This strain was characterized genomically as type B:2,5. This Canadian case is thought to be the first officially reported occurrence of HS in Canada, and like our case 1, was identified in 2020.^16^ Two B:3,4 strains were collected from cattle and cervid species in New Zealand in 2022,^21^ and these strains also clustered with the North American strains. The phylogenetic tree suggests an evolutionary similarity of our case strains to the New Zealand strains, but it should be noted that the strains originated contemporarily with the New Zealand strains from 2022.^21^

Pangenome analysis suggests some regional relatedness of the strains. Clade 2 consists of 4 North American and 2 New Zealand strains; of those, 5 were B:3,4 strains and 1 was a B:2,5 strain. Clades 1, 3, and 4 are comprised of classical B:2,5 HS strains. Clade 1 consists of strains from the United States and China. Clades 3 and 4 originate from Asia. CP017961 was isolated from a bovid in Iran in 1936; this strain was used to develop a vaccine for HS.^37^

New MLST profiles were assigned in the PubMLST database for P1669, and cases 1 and 2, supporting the evidence that these strains are dissimilar from those circulating globally. Most strains in our study (11 of 17) were RIRDC MLST ST122 (clonal complex ST122), a finding consistent with HS cases reported in PubMLST. RIRDC MLST profiles in the PubMLST database were reviewed on 2024 April 22 to determine the origins of similar strains. The New Zealand accession CP133810 is the same sequence type (RIRDC ST62) as other strains previously isolated in New Zealand.^27^ The second accession from New Zealand, CP133809, is consistent with a sequence type (RIRDC ST64) originating from a turkey in Denmark in 1989.

There are several limitations to our study. Our genomic analysis simply compared shared genes but did not assess which genes make the type B:3,4 strains different from circulating B:2,5 strains. Very few whole-genome sequences of HS-causing strains of *P. multocida* exist publicly.

Interestingly, HS has re-emerged in several developed countries in recent years. Germany had no reported cases of HS from 1986 to 2010 and has since experienced several outbreaks.^24,38^ Spain,^32^ Denmark,^1^ and Hungary^26,41^ have also seen re-emergence in both wild and domestic animal species. The reasons for this re-emergence are unclear. Outbreaks of HS have historically been associated with wet environmental conditions,^15,44^ and global climate change may be a factor. Accessibility to technologies such as the capsular typing PCR or whole-genome sequencing may also have made detection of HS more attainable in recent years. MALDI-TOF MS may further facilitate the detection of HS-causing strains and could be an important rapid screening tool for suspect cases.^25^ If similar trends of re-emergence are observed in the United States and Canada, diagnosticians would be well advised to acquaint themselves with the presentation of this disease. Bovine cases of fibrinosuppurative serositis, particularly pericarditis with or without myositis, that lack significant bronchopneumonia and have widespread hemorrhage from which *P. multocida* is isolated, should be regarded as suspicious for HS. Capsular typing of strains from such cases may be warranted because colony morphology of HS-causing strains of *P. multocida* are not visually different from other capsular types and, therefore, would not be routinely characterized further beyond genus and species of the bacterium.

Future areas of study should focus on determining potential HS reservoir species that might facilitate the spread of disease. As new strains are discovered, sequences should be shared publicly to allow further study of these pathogens. These strains are of importance, particularly in developing countries, due to the dependence on susceptible species economically and as a food source.^15^ Further study of HS-causing strains may facilitate development of better intervention and prevention strategies for this devastating disease.
